# Mixed methods assessment of impact on health awareness in adult childhood cancer survivors after viewing their personalized digital treatment summary and follow-up recommendations

**DOI:** 10.1186/s12885-021-08051-9

**Published:** 2021-04-01

**Authors:** Helena M. Linge, Cecilia Follin

**Affiliations:** 1 Department of Clinical Sciences Lund, Pediatrics, Faculty of Medicine, Lund University, Lund, Sweden; 2 Department of Clinical Sciences Lund, Oncology, Skane University Hospital, Lund University, Lund, Sweden

**Keywords:** Digital, Treatment summary, Ehealth, Late effects, Childhood cancer, E health literacy

## Abstract

**Background:**

The survival rate after childhood cancer has improved to 80%. The majority of childhood cancer survivors (CCS) will experience late complications which require follow up care, including access to their individual cancer treatment summary. The need to understand CCS needs and preferences in terms of ways to receive information e.g. digitally, becomes important. This study aims to through a mixed methods approach a) examine how CCS’ health awareness was impacted by viewing their personalized digital treatment summary and follow-up recommendations, b) explore E health literacy, and c) determine self-reported survivorship experiences and health care usage.

**Methods:**

Survivors with a recent visit to the Late effects clinic were eligible for the study (*n* = 70). A representative sample of primary diagnoses were invited (*n* = 28). 16 CCS were enrolled. Recent medical visits, e health literacy and impressions of the digital treatment summary were assessed by a survey in conjunction with viewing their digital treatment summary on a computer screen. Their experience of reading and understanding their digital treatment summary in the context of their health related survivorship experiences were assessed in focus groups. The transcribed data was analyzed with conventional qualitative content analysis.

**Results:**

The self-reported medical problems largely reflected that, only 6,3% reported no cancer-related reasons for seeking medical attention. Of the medical specialists, the primary care physician was the most frequently visited specialist (68.8%). High E health literacy was not associated with treatment features but with educational level (*p* = 0.003, CI: 3.9–14.6) and sex (*p* = 0.022, CI: − 13.6- -1.3). All survivors graded the digital treatment summary above average in terms of being valuable, agreeable and comprehensive. The focus group interviews identified three themes: 1) The significance of information, 2) The impact of awareness; and 3) Empowerment.

**Conclusions:**

Reading the treatment summaries furthered the survivors understanding of their health situation and consequently aided empowerment. A digital treatment summary, provided by knowledgeable health care professionals, may increase the self-managed care and adherence to follow-up recommendations. Further insights into e health literacy in larger samples of CCS may determine to what extent health-related information can be communicated via digital resources to this at risk population.

**Supplementary Information:**

The online version contains supplementary material available at 10.1186/s12885-021-08051-9.

## Background

The survival rate after childhood cancer has improved markedly and today more than 80% of patients with a paediatric malignancy will become 5-year survivors [1]. Currently, one in every 1000 young adults in developed countries is a childhood cancer survivor (CCS) [2]. However, it has become evident that many CCS suffer from medical, cognitive and psychosocial late complications. Reports indicate that 70–90% of CCS will experience one or several late complications due to cancer treatment, resulting in excess morbidity and mortality compared to gender matched controls [3, 4]. These complications require regular follow-up care to preserve health, improve quality of life and increase empowerment [5]. In addition to the follow-up care at the hospital and a long-term relationship with qualified health-care professionals, the survivors need education about their diagnosis, treatment history and follow-up plans, including access to their individual cancer treatment summary.

75% of the survivors are unaware of potential late complications after cancer treatment during childhood [6]. Information about potential risks of complications is essential in order to attend follow-up care and to engage in healthy behaviour [7]. Further, survivors themselves report a lack of adherence to recommended screening programmes, based on evidence-based guidelines, and medical examinations as they enter adulthood, which places these survivors at a particularly high risk for chronic conditions [8]. Having a holistic approach in the meeting with the survivors is valuable as it has been shown that the survivors require knowledge and support in order to handle and understand their complex situation [9].

The success of the model of follow-up care depends on the survivors’ attendance, which is associated with the survivors’ preferences and understanding of their situation [10, 11]. CCS may be at risk for not having access to and understanding the cancer treatment summary due to limited access to information, their young age at cancer diagnosis and the influence of parental decision-making. Several studies have shown that survivors are unaware of their risk for complications and are inadequately prepared to seek out necessary survivorship care [12, 13]. There has long been a shortage of information about survivors’ experiences of follow-up methods and their specific information needs [14, 15]. Finding different options to improve the survivors’ knowledge of their history, and also increasing compliance with follow-up recommendations, are a challenge for late effect clinics. Health-care providers need more knowledge about how the survivors experience having access to their individual cancer treatment summary and follow-up recommendations, in order to be able to increase awareness and optimize and adapt the information to the survivors’ needs. Digitalization and direct access represents one such option, which could lead to a higher degree of self-managed health [16]. This study aims to 1) examine the impact of viewing and understanding a personalized digital treatment summary and follow-up recommendations on CCSs’ health awareness, 2) explore E health literacy among the survivors and 3) determine self-reported survivorship experiences and health care visits. We used a mixed methods approach in order for the results to show a broader representation than use of only one of the methodologies would allow.

## Method

### Digital treatment summary and follow-up recommendations

The launch of a digital treatment summary has been described [17]. In brief, registry data [18] containing treatment history pertinent to medical follow-up is presented in the format of a report. The report contains patient information (i.e. date of birth, date of primary diagnosis, survival time), and information on the primary diagnosis (i.e. classification according to ICCC, stage, anatomical location, SNOMED and ICD10). Information on the four different treatment modalities surgery, radiation therapy, chemotherapy and stem cell transplantation are depicted in tabs in the digital treatment summary. Cumulative doses of chemotherapeutic agents, radiation doses organized according to anatomical site of treatment, and details of radicality or removal of hormone producing organs, are provided. The digital treatment summary shown with frames in red, orange and gray was accompanied by general follow up recommendations relevant to each survivor treatment profile. The presented material did not contain individualized instructions or prompts for direct medical visits. The analog treatment summary that the CCS had received prior to this study (see inclusion criteria) contained the same medical information but was constructed as a list with a black- and-white lay-out.

### Recruitment and participants

The inclusion criteria were: 1) diagnosis with childhood cancer according to ICCC under the age of 18 years; and 2) a visit to the late effect clinic at the University Hospital in Lund, Sweden, 1–6 months prior to the present study, and 3) informed consent. CCS with cognitive deficits; and CCS who had attended the clinic more than 6 months ago were excluded from the study. 70 survivors were identified as eligible. Twenty-eight survivors were invited to participate. We sought to assemble a representative sample (diagnosis, gender, age,) of the childhood cancer survivor population in the study cohort. They were sent a letter with information about the study which included an informed consent form to be signed and returned if they were willing to participate. Two weeks after sending the letter, one of the authors (C.F.) phoned the patients to provide further information about the study and to ask if the CCS were willing to participate. Nine survivors declined participation due to a lack of time or lack of interest, and three failed to come to the meetings. A total of 16 survivors participated in the present study.

### Study design and data collection

**Fig. 1:**
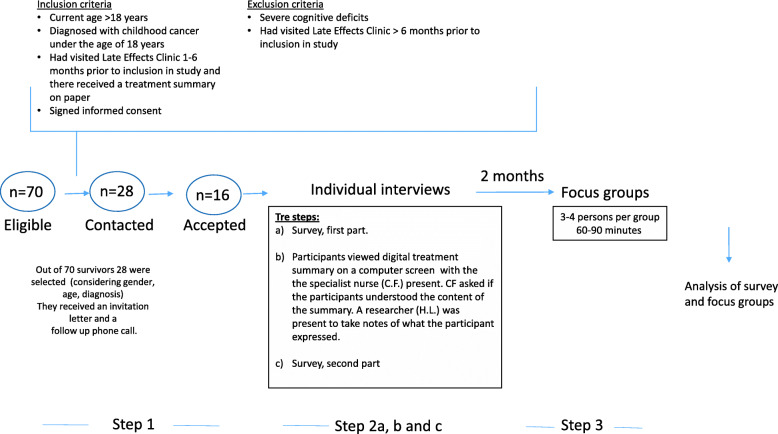
Schematic figure of study design

During the individual interview (Fig. 1 step 2a), each participant completed the first part of the survey. The survey was adapted from McClellan et al. [19] and supplemented with 4 items about evaluation adapted from other digital health tools [20, 21], and 6 queries regarding e-health literacy [22]. Each item in the eHEALS uses a 5-point scale to answer each question with response options ranging from “strongly agree” to “strongly disagree”. After viewing the personalized digital treatment (Fig. 1 step 2b), the participants were asked to comment on the legibility, lay-out and understanding of the digital presentation summary as it was presented on a computer screen. Then the second part of the survey was completed (Fig. 1 step 2c).

Two months after the individual interview, participants were gathered to address survivorship issues and their experience of accessing the digital treatment summary in focus groups (Fig. 1 step 3). A semi-structured interview guide was used to facilitate focus on the aim of the study [23]. The moderator’s primary focus was on helping the respondents keep to the topic. The interview started with an open question asked by the moderator (H.L.): “*How would you describe your experience of reading and understanding your digital treatment summary?”.* The observer (C.F.) assisted by asking probing questions (see Supplement) and taking notes and by having follow-up discussions that needed clarification during the interviews. The observer concluded the interview by giving a short summary thereof. The focus group interviews (60–90 min in length) were conducted in a separate room at the university hospital library. The authors considered a representative inclusion of diagnoses among the survivors when assembling the groups. The survivors also had the choice to request a specific interview occasion which suited their schedule to attend.

### Data analysis

The results of the survey (Fig. 1 step 2) were summarized using descriptive statistics after the final focus group occasion. Multiple linear regression was used to determine the correlation between the eHealth literacy score and the variables “time since diagnosis” and “radiotherapy to CNS”. SPSS version 26.0 was used for the quantitative analysis. We used a truncated eHEALS item set of 6 items. The reason for truncation was the seeming redundancy of the items “I know where to find helpful health resources on the Internet” and “I have the skills I need to evaluate the health resources I find on the Internet” after translation into Swedish. The score could range between 6 and 30 points. We followed the approach of Milne et al. to show how many CCS were considered “as high responders” [24]. The approach is in line with Park et al. who, on the 8 item scale set the cut-off point at 24 points [25]. For the 6 item scale, we used 20 points as the cut-off.

The qualitative assessment (Fig. 1 step 3) was conducted without the support of any specialized software and as described before [9]. In short, the interviews were recorded as a data file and transcribed *verbatim*. A conventional qualitative content analysis was used to analyze the data [26]. The two authors who conducted the interviews analyzed them all. The analysis was carried out as follows: firstly the text was read as a whole, to gain a general understanding. Secondly, the text was read again, word for word, with a focus on identifying codes that captured key concept and thoughts. As the analysis proceeded, labels for codes emerged that were reflective of more than one key thought, and together the codes resulted in the initial coding scheme. Codes were then sorted into categories and subcategories. The authors then discussed the categories together. Themes were agreed upon and categories named and sorted under the corresponding theme.

## Results

The characteristics of the study group (*n* = 16) which aimed at a broad representation of diagnoses, are shown in Table 1. The survivors’ median age at interview was 39 years and they were interviewed in median 31.5 years after cancer diagnosis. 9 survivors were treated with radiotherapy in combination with chemotherapy and 7 survivors with chemotherapy alone. Further details on participant demographics is shown in Table 1.

**Table 1:** The characteristics of the survivors (*n* = 16)

Sex (male/ female)	6/10
**Current age (years)** Median (range)	39 (23–56)
**Highest completed level of education**	Elementary school
	1/16
	Secondary school
	10/16
	University
	5/16
**Current level of employment**	Employed
	11/16
	Short term disability compensation
	1/16
	Long term disability compensation
	3/16
	Unemployed
	1/16
**Age at diagnosis (years)** Median (range)	7.19
	+/− 4.7 (2-17)
**Time since diagnosis (years)** Median (range)	31.5 (20–50)
**Diagnosis**	ALL 6
	CNS-tumours 4
	Hepatoblastoma 1
	Lymphoma 2
	Sarcoma 1
	AML 2
**Any RT**^**a**^ **(y** ^**b**^ **/total)**^**c**^	9/16
**RT**^**a**^ **to CNS (y** ^**b**^ **/total)**^**c**^	8/16
**Chemotherapy**	16/16
**Surgery**	5/16
**Stem cell transplantation**	1/16

CNS = central nervous system. ^**a**^ radiotherapy; ^b^ yes; ^**c**^ total number

### Survey results

Regarding their respective health status (Fig. 1 step 2a) the majority of participants (15/16; 94%) graded their health/well-being as “fair” or greater. Most participants reported that they felt that their needs had been taken seriously by the health care provider (less than fair [2], fair [1], good [7], excellent [4], no replies [2]). 12 of the 16 participants (75%) had at some point in their lives been in contact with health care providers for reasons that they believed were linked to their childhood cancer treatment. In the 24 months prior to the study, the group had on average had 2.56 +/− 1.59 (median 2) health care visits. The type of medical specialists which the participants had visited in the last 24 months are shown in Table 2, panel A. The most visited specialist was the primary care provider (PCP) (68,8%) and the least visited was psychiatrist and gastrointestinal specialists (both 0 visits). The detailed self-reported medical issues and emotional experiences are shown in Table 2, panels B and C.

**Table 2:** Self-reported medical visits, physiological problems and emotional experiences

A. Visit to a clinical specialist in the last 24 months	n ^**e**^	%	B. Self-reported physiological problems	n	%	C. Emotional experiences	n	%
PCP ^**d**^	11/16	68.8	Memory	5/16	31.3	Fear of cancer reccurence	8/16	50
Counselor	4/16	25.0	Learning	5/16	31.3	Feeling vulnerable	3/16	18.8
Chiropractor	2/16	12.5	Attention	4/16	25.0	Diminished physical strength	4/16	25.0
Physiotherapist	2/16	12.5	Growth hormone deficiency	7/16	43.8	Lacking enthusiasm	3/16	18.8
Psychologist	1/16	6.3	Weight	4/16	25.0	Overwhelming enthusiasm	0/16	0
Cardiologist	4/16	25.0	Fertility	6/16	37.5	School troubles	4/16	25.0
Otolaryngologist	3/16	18.8	Physical activity	6/16	37.5	Fear of death	4/16	25.0
Endocrinologist	6/16	37.5	Liver function	0/16	0	Change of body appearance	4/16	25.0
Pulmonologist	1/16	6.3	Thyroid gland	2/16	12.5	Relationships changed	4/16	25
Diabetes team	2/16	12.5	Pulmonary function	2/16	12.5	Attention deficits	4/12	25
Nephrologist	1/16	6.3	Cardiac function	3/16	18.8	”Impossible to work”	3/16	18.8
Neurologist	2/16	12.5	Hearing loss	6/16	37.5	Feeling of gratitude	5/16	31.3
Psychiatrist	0/16	0	Gastrointestinal function	1/16	6.3	”No one understands me”	5/16	31.3
Gastrointestinal specialist	0/16	0	Depression	3/16	18.8	Private economic burden	4/16	25
Other medical specialist	2/16	12.5	Anxiety	3/16	18.8	Difficulties with the public employment service	4/16	25
			Fatigue	5/16	31.3	Difficulties with the National Insurance Agency	1/16	6.3
			Secondary malignancy	2/16	12.5			
			Other	2/16	12.5			
			None	1/16	6.3			

^**d**^ primary care provider; ^**e**^ number;

Eight participants (50%) reported having received a treatment summary on paper, 6 (37.5%) reported not received and 2 (12.5%) were unsure. 14 reported being aware (*n* = 7), or partially aware (n = 7) of their personal follow-up recommendations, whereas 2 reported that they were unaware. During the present study, 4 (25%) had planned to contact the late effect clinic.

**Fig. 2:**
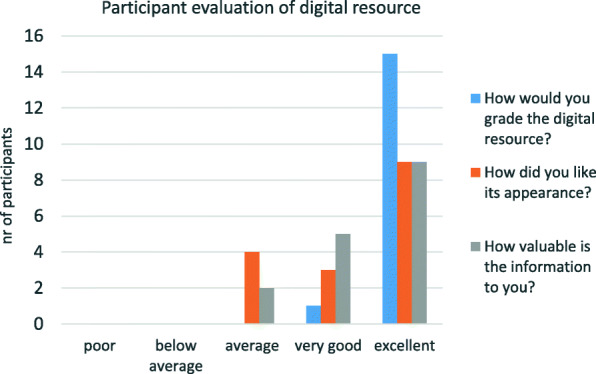
Participant evaluation of digital resource

The overall response to the digital treatment summary (reported in step 2c, Fig. 1), its appearance, and the value of the presented information, was positive with 100% of the participants grading it “very good” or “excellent” (Fig. 2). The participants deemed the appearance agreeable and reported that the content held a high value to them. Of the 16 participants, 6 expressed that they had suggestions for changes, whereas 10 did not. The changes that were suggested included explanations of medical terms and abbreviations primarily regarding the diagnosis, e.g. SNOMED, ICD10 and “recurrence”. It was expressed that the participants wanted headings and fields to be shown even when a particular treatment module was not a part of their medical history. Two participants expressed that personalized calls to action were lacking. The recommendations were too general in their opinion. They expressed doubts about which parts of the recommendations really applied to them. Two participants reported they would view the summary once per month, and 2 reported once every 6 months. The majority (12/16) of participants stated they would review the treatment summary if and when a need would arise.

**Fig. 3:**
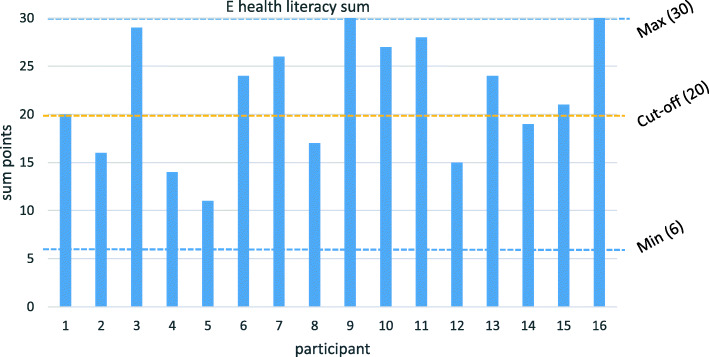
Total ehealth literacy score

With regards to the E health literacy scores (reported in step 2c, Fig. 1), 9/16 participants (56%) had a score higher than 20 (dotted line, Fig. 3) where the maximum was 30 points. 7 participants reported agreement with the statements to the degree of very well or excellent (scores 4 or 5) on 5 or more of the 6 items. Out of the 6 items, the statement which received the highest average score of the participants was “I know how to find helpful health resources on the Internet” (4.06). The statement with the lowest score was “I know what health resources are available on the Internet” (3.06).

The E health literacy sum did not correlate with “age at diagnosis”, “time since diagnosis”, or “CNS radiotherapy”. It was however significantly correlated with sex (*p* = 0.022, CI: − 13.6- -1.3) and the level of education (*p* = 0.003, CI: 3.9–14.6).

### Focus group interview results

The qualitative section of the study (Fig. 1, step 3,) gathered more extensive data on the survivorship experiences from the participants in their own words. The time (2 months) that had passed between survey and viewing of the digital treatment summary (Fig. 1 step 2) - to focus group participation (Fig. 1 step 3) was chosen to allow the participants enough time to consider the digital treatment summary and how the information had affected them. In the analysis of the focus group interviews, the following themes and categories were identified. Quotes are shown as examples.

### Theme 1: The significance of information

#### Category: Access

The survivors highlighted the value of having access to an extensively detailed treatment summary stating the risk of potential complications. They expressed realizing that having this knowledge could have an impact on their future health. The survivors highlighted the feeling of relief when they read the treatment history and learned how this information could become available to them digitally. They expressed that they with access to a treatment summary no longer had to remember the details, nor repeat their history in contact with health care. This resulted in a feeling of safety.

“It is always available; I don’t have to search for it.”

Interview 3.

“To not have to search for the information, to not have to go on a wild goose chase to find a doctor when I have questions- which makes things easier for health care as well- to not have to hunt down the information”.

Interview 3.

In contrast to having the information the participants expressed that not knowing may lead them to think that they can ignore the risk of complications.

If it doesn’t mention particular risks, you are inclined to think you are out of the woods. Evidently there are multiple things to be aware of.”

Interview 2

” Personally, I want it to state everything. Otherwise it may lead you to think you are not at a particular kind of risk, simply because the information is lacking. “

Interview 2

#### Category: Trustworthiness

Further, the participants experienced trust when gaining access to data provided from paediatric oncology professionals and they stressed the importance of reliability of the information in order to feel secure.

” It is better to have a proper informative format, with proper sources and correct information. Much better than going to Google, or finding Wikipedia where sources are missing. A trustworthy source is important.”

Interview 1.

The survivors’ experience of unclear or incorrect information, presented to them in the past, had resulted in a feeling of unsafety. Being offered guided information, compared to unguided (Fig. 1, step 2b), led them to understand their cancer history and how this could influence their future life situation.

“It is always easier when there is someone to talk to. I think it is better to receive it together with someone else, like this interview, instead of receiving it in the mail.” Interview 1

“It can be interpreted in so many different ways, if you receive it on your own. I feel much more secure knowing that I can call the Late Effects clinic.”

Interview 1.

Although there was a desire for personalization of the information and a belief that this could make the treatment history more useful, the survivors also recognized the complexity of having too many details presented. Further, they stressed a feeling of doubt about the individual data collection, including at what level of detail it should be presented and the resources this would require.

” I’d like it more personalized. But that may require many different pieces of information to be retrieved and put together. I don’t know if it is even possible.”

Interview 2.

#### Category: The timing of information

The participants highlighted different aspects of when the information about potential risks should be provided. One example was receiving honest and detailed information at the end of treatment when they were still very young. Further, they also expressed that the information could also be spaced in time to fit their particular stage of life. The survivors experienced a positive timeliness link between receiving the information and coping with the risks or awareness of complications.

“It makes the most sense to know all the risks from the beginning. ”

Interview 2

“But some side effects may not be evident until after 20 years- if so, you may not want to worry those people.”

Interview 2

“It came as a shock. It worried me, and I thought -Is this really a complication of the irradiation and medication? No one had told me I could go deaf.”

Interview 3.

### Theme 2: The impact of awareness

The participants experienced advantages, as well as disadvantages of knowing and not knowing about complications and risks of complications. They were left with a feeling of confusion and imbalance when the realized that they had poor knowledge of late effects and were unprepared. Absence of information resulted in worry among the survivors and, in the void of information, they had handled their situation on their own.

“I didn’t tell my children that I had been ill. Because I was afraid that they would think that they would get sick. I’ve kept it a secret this entire time.”

Interview 1

“It is really tough. The cancer diagnosis is so stigmatizing. When you tell someone you’ve had cancer, they look at you as if you’ve had one foot in the grave. ”

Interview1.

Receiving information about complications, as well as future risks of developing complications, was an experience resulting in new insights, coping strategies and an increased level of confidence. Some survivors brought up that they were not sure about knowing too many details as it may result in negative thoughts about the future.

“I keep the information at the back of my mind but try not to think about it on an everyday basis.” Interview 2

“Knowing is a good thing. It creates attentiveness. But it can also be hard knowing. It is a balance.”

Interview 1.

### Theme 3: Empowerment

Receiving and understanding their digital treatment information and follow-up recommendations led the survivors towards an increased health-related self-confidence. They expressed that preventive measures in terms of lifestyle choices were closer at hand after they had become aware of their potential risks. The survivors’ desire of more information, including their interest in understanding the nuances of the treatments and medical follow-up, showed their willingness to take more responsibility for, and to play an active role in their own health situation. This can be interpreted as an expression of empowerment among the survivors.

“Knowledge is power- the more you know the more you can be attentive of signs or symptoms”

Interview 1

“I can adjust my habits in order to reduce the risks, if I am in a high risk group.”

Interview 3.

## Discussion

The present study focused on the reactions of CCS with a representative spectrum of primary diagnoses and with a relatively long follow-up time, to a digital treatment summary. Using mixed methodology, we sought to determine the self- reported health situation of 16 CCS and how they experienced receiving digital access to their treatment summary and risk group-based follow-up information. As expected, and in concordance with previously recognized late effects [27], we found that the following somatic late effects were reported to a high degree from the participants: growth hormone deficiency, fertility issues, physical activity issues, hearing loss, and overweight. The impaired cognitive functions e.g. problems with memory, learning, attention and also fatigue were reported by 25% or more of the survivors. Among the emotional experiences, fear of cancer recurrence was the most widely reported. Only 6.3% experienced no late effects. This is in agreement with that 80–90% of CCS will experience late complications [4].

The current study points to important aspects of how it would be experienced by CCS if they received their treatment summary in a digital format in concordance with, or separation from, the late effects clinic. In general, the CCS were satisfied with the content of the received information, which they expressed held a high value to them. The results from the focus group interviews demonstrate the strength of having access to the digital information but also the desire to have it delivered and interpreted to create understanding together with a trusted party i.e. the late effects clinic professionals. A Norwegian study recently investigated the preferences for follow-up care after childhood lymphoma [28]. The preference in that study was in favor of professionals with necessary knowledge over PCPs, nurse practitioners, internet, self-help or peer help. Ramsay et al. (2018) determined the follow up care preferences of cancer survivors diagnosed between ages 15–39 and found that a continued relationship with their oncologist was preferred [29]. In our study the PCP was the most visited health care representative. This points to a discrepancy in the delivery of care. As the awareness of the Late Effects clinic increases over time this discrepancy may be diminished. It is interesting that although receiving a treatment summary was an inclusion criterion for enrollment in our study, only half of the participants replied affirmatively to this question. This may indicate lack of clarity in the health care situation, a stress-related memory loss which may be specific or unspecific for the CCS. It suggests a need for novel approaches in delivering the information. Thus, our results support that providing follow-up care at the Late effects clinic in combination with access to the digital summary and care plan could be a way to improve the survivor’s health awareness, survivor-provider communication and adherence to follow-up recommendations.

When evaluating different models on how to present a care plan it is crucial to take into account the survivors’ preferences and experiences. Previous studies show the survivors’ experiences of having their complications poorly explained [30] and also how they experienced unanswered questions after a visit to the clinic [31]. The participants in our study expressed similar experiences from their past. There are challenges with providing the survivors with information about possible complications which may result in a feeling of vulnerability. We noted this in the focus groups. In contrast, we also noted that the reading and understanding their treatment summary and follow-up recommendations through digital channels led to empowerment among survivors. However, the digital treatment summary and follow-up recommendations provide a platform to educate and support the CCS during adult life to engage in self-care. Indeed, in the present study we report on the survivors’ desire of information, including their willingness to play an active role in their own health-situation. This is in line with McClellan et al. (2013) reporting a need of access to information among CCS, in particular in survivors who have received intense treatment [19].

The eHEALS items measures an individual’s knowledge of health information resources on the Internet and more specifically, the self-perceived confidence in their ability to locate, evaluate, and use this health information to make informed health decisions [22]. To the best knowledge of the authors, E health literacy has not been assessed for childhood cancer survivors before. It has however been assessed in adult lung cancer survivors (*n* = 83; median age 71 (range 44.89)) [24]. The Milne study grouped participants into high self-perceived eHealth Literacy (scored a 4 or 5 on at least 5 out of the 8 eHEALS items) and low self-perceived eHealth literacy. In our study, 7/16 were classified as high self-perceived e health literate, reporting a score of 4 or 5 on 5 out of the 6 items used in the current study. Despite the relatively small sample size and the truncated eHEALS, we found that the total eHEALS score correlated with sex and level of education but not time since diagnosis, or whether patients had received radiation therapy to the CNS. The correlation to overall educational level is consistent with the findings of others [24, 32]. Significant associations between eHealth Literacy and female sex has previously been described in studies of healthy US adult populations [25, 33] and has been suggested to associate with the role of being the primary health information seekers in their families. Awareness about the level of eHealth Literacy may help guide health care providers and policy makers to what extent health-related information can be communicated via digital resources to CCS. Measuring and reporting e health literacy is hence a step towards reaching a shared responsibility for survivorship after childhood cancer.

The study should be viewed in light of its strengths and limitations. The study sample is small but representative in terms of sex, age, and primary cancer diagnosis; and should be viewed in the current setting of proximity to a newly established Late Effects clinic at the Skåne university hospital in Lund, Sweden. The small sample size may have reduced the ability to detect associations beyond what we have reported. The author’s differences in clinical experience with CCS enables interpretation of the qualitative results from different perspectives, which is a strength of the study. The mixed methods approach enabled us to record self-reported variables together with a spectrum of views on survivorship following pediatric malignancies. Although translated and truncated, the results of the eHealth Literacy assessment are supported by previous studies. Future studies with larger sample sizes possibly from different geographical settings, and a strict translation process of the eHEALS items are needed to fully elucidate the standing of eHealth Literacy and corroborate the findings. What the participants reported are perceived skills and health care visits. The study did not check for actual skills or verify the reported health care visits.

In summary, we assessed the self-reported health status and e health literacy of 16 CCS and followed their experiences through focus group interviews to explore how a digital treatment summary may contribute to awareness and empowerment among the survivors. We report that a digital summary together with follow up recommendations delivered by knowledgeable professionals has the potential to improve the survivor’s health awareness, survivor-provider communication and possibly in the long term also adherence to follow-up recommendations to promote CCS long term health.

## Conclusion

In conclusion, CCS with a representative spectrum of primary diagnoses and a relatively long follow up time, were impacted by viewing and understanding the digital treatment summaries. Reflecting on the information at the emotional level furthered the survivors understanding of their health situation and consequently aided empowerment. Further insights into e health literacy in larger samples of CCS will help determine to what extent health-related information can be communicated via digital resources to this at risk population. Digital treatment summaries delivered by knowledgeable health care professionals could support continuous health surveillance and promote the patient-health care-shared responsibility of medical follow up after childhood cancer.

## Supplementary Information

**Additional file 1.**

## Data Availability

The datasets used and/or analyzed during the current study are available from the corresponding author on reasonable request.

## Abbreviations

CCS: childhood cancer survivor
CNS: central nervous system
RT: radiotherapy
PCP: primary care provider
N: number
