# Supplementary material for: Mixed methods assessment of impact on health awareness in adult childhood cancer survivors after viewing their personalized digital treatment summary and follow-up recommendations
Source: BMC Cancer. 2021 Apr 1;21:347. doi: 10.1186/s12885-021-08051-9 (PMC8017604; doi:10.1186/s12885-021-08051-9)
Supplement: Supplementary file 1 — Additional file 1. [file 12885_2021_8051_MOESM1_ESM.docx]

**Focus group interviews**

**Interview guide**

CF informed and clarified the following before the focus group interview started

- The purpose of the study
- The focus is on the digital presentation of the treatment summary and the experience of reading and understanding it.
- All participants will be able to speak, and speak without interruption
- If I interrupt, it is because we have drifted away from the topic
- It is important that you say exactly as you feel and what you experience
- Everyone has a different treatment background, so lets be respectful of eachothers differences
- The focus group inetrview will last no more than 2 hours.

Main question:

HL:

How would you describe your experiences of the digital presentation of your treatment summary from being treated for cancer in childhood?

Areas we would like to illuminate during the interview:

The experience of the digital presentation of your treatment summary

- What do you think the purpose is when viewing your treatment summary and the potential risks you might have for late effects?
- What is your purpose of reading the summary?
- Is there something that you experience as extra important?
- How does viewing and understanding the information on your treatment and possible late effects affect you?
- Do you want someone to be available after you have read your treatment summary? A nurse, a doctor? How can the designated nurse be of support here?
- How should the treatment summary be presented? Digitally or on paper?

The experience of learning about late effects

- How would you describe your knowledge about the risk of being affected by late effects?
- Can you give examples on how you have received information about the late effects?
- Do you feel that you want to know what risks there are? Why would you like to know/not know?

The focus group interview is ended by:

CF summarized how we have perceived the patients’ experiences and asked:

- Do you agree?
- Is there something you would like to add?
